# Ion Implantation Combined with Heat Treatment Enables Excellent Conductivity and Corrosion Resistance of Stainless Steel Bipolar Plates for Hydrogen Fuel Cells

**DOI:** 10.3390/ma17040779

**Published:** 2024-02-06

**Authors:** Ruijuan Wang, Li Ding, Yong Pan, Xin Zhang, Meng Yang, Chengfei Zhu

**Affiliations:** 1 College of Safety Science and Engineering, Nanjing Tech University, Nanjing 211816, China; 202161201102@njtech.edu.cn (R.W.); xinzhang@njtech.edu.cn (X.Z.); 2 School of Automotive & Rail Transit, Nanjing Institute of Technology, Nanjing 211167, China; 3 College of Materials Science and Engineering, Nanjing Tech University, Nanjing 211816, China; yangmengyy@njtech.edu.cn (M.Y.); njtechzhucf@163.com (C.Z.)

**Keywords:** 316 L stainless steel bipolar plate, ion implantation, heat treatment, interfacial contact resistance, corrosion resistance

## Abstract

316 L stainless steel is an ideal bipolar plate material for a proton exchange membrane fuel cell (PEMFC). However, the thickening of the passivation film on the stainless steel surface and the dissolution of corrosive ions during operation will affect the durability of the PEMFC. Herein, a heterogeneous layer is prepared on the surface of 316 L stainless steel through dual ion implantation of molybdenum ion and carbon ion combined with heat treatment to promote the corrosion resistance and conductivity of the bipolar plate. The ion implantation technique resulted in a uniform distribution of Mo and C elements on the surface of 316 L stainless steel, with a modified layer depth of about 70–80 nm. The electrical conductivity of the ion implanted samples was significantly improved, and the interfacial contact resistance was reduced from 464.25 mΩ × cm^2^ to 42.49 mΩ × cm^2^. Heat treatment enhances the surface homogenization, repairs the defects of irradiation damage, and improves the corrosion resistance of stainless steel. The corrosion current density of (Mo+C)-600 samples decreased from 1.21 × 10^−8^ A/cm^2^ to 2.95 × 10^−9^ A/cm^2^ under the long-term corrosion condition of 4 h. These results can provide guidance for the modification of stainless steel bipolar plates.

## 1. Introduction

Given the depletion of non-renewable energy sources and escalating environmental pollution, fuel cells are anticipated to assume a pivotal role in the foreseeable future. The proton exchange membrane fuel cell (PEMFC), owing to its exceptional energy conversion rate, zero emissions, and rapid startup at room temperature, has emerged as one of the primary power sources for electric vehicles [1,2]. The bipolar plate (BP) is the core component in a PEMFC, and accounts for 70% of the weight, almost all of the volume, and 30–50% of the production cost of the entire cell stack [3,4]. Consequently, bipolar plates play a crucial role in PEMFC battery packs by providing support to the membrane electrode assembly, facilitating gas conduction, current collection, and water drainage [5]. Henceforth, it is imperative for bipolar plates to meet requirements such as excellent corrosion resistance, high electrical conductivity, relatively low cost, and superior strength while maintaining compact dimensions [6,7,8]. Currently, the commonly used materials for bipolar plates in hydrogen fuel cells can be categorized into three main groups: graphite plates, composite plates, and metal plates [9,10]. Metal materials have excellent mechanical strength, good electrical conductivity and high corrosion resistance, vibration durability and other advantages. 316 L stainless steel metal materials in particular, with high strength and low-cost advantages, are considered one of the ideal materials for bipolar plates.

Hodgson et al. [11] showed that 316 L stainless steel exhibited poor performance in fuel cells due to increased contact resistance caused by the thickening of the surface passivation layer. Hentall et al. [12] found that Untreated stainless steel surfaces showed an increase in contact resistance due to the formation of the passivation layer in the fuel cell environment through the study of 316 L stainless steel. 316 L stainless steel plates lack corrosion resistance in acidic and electrochemical environments [13,14]. The presence of corroded metal ions contaminates the electrolyte film and poisons the electrode catalysts, thereby deteriorating PEMFC battery pack performance [15]. Therefore, surface modification is necessary to improve both electrical conductivity and corrosion resistance of stainless steel bipolar plates in PEMFC. Lee et al. [16] chromium plated stainless steel and then thermally nitrided it to form a Cr_2_N layer on the surface to improve the corrosion resistance of 316 L stainless steel to meet the requirements of fuel cell bipolar plates. Viviente et al. [17] investigated the injection of extremely high doses of C^+^ (in the ionic range of 10^18^) into martensitic stainless steel (440 C) and Ti6Al4V substrates, revealing the presence of carbide and C-C bonds, as well as enriched compounds such as carbides and oxides, on the surface of these carbon layers. The titanium alloy samples exhibited higher carbide contributions compared to AISI 440 C steel samples due to their greater Ti to C ratio. Liang et al. [18] investigated the deposition of Ti+C and Mo+C on an H 13 steel surface using a filtered vacuum arc plasma deposition (FVAPD) system, resulting in thin binary layers of Ti, C, and Mo. The corrosion properties of these coatings were evaluated in acetic acid, sodium acetate solution, or sodium chloride solution by employing the three-electrode potential kinetic polarization technique. The results indicated that the binary coatings prepared through this method exhibited excellent corrosion resistance. Kiniger et al. [19] explored the sputter deposition of a 100 nm-thick molybdenum (Mo) interlayer between copper and carbon, which after heat treatment at 600 °C produced high carbon signal throughout the entire Mo interlayer forming a Mo_2_C substance. Kim et al. [20] studied various chemical and heat treatments on 446 M ferritic stainless steel to investigate their effect on ICR and corrosion resistance enhancement. The combination of chemical and heat treatments not only improved the corrosion resistance but also reduced ICR. Li et al. [21] examined the impact of two different heat treatments on 316 L SS’s corrosion behavior in a simulated cathodic environment. The coating method makes the interface between the modified layer and the substrate obvious, and it easily falls off through external force. Researchers and scholars have used heat treatment methods or coating and heat treatment combined method in 316 L stainless steel safety performance research, but there is still the problem that corrosion resistance and electrical conductivity cannot simultaneously meet the requirements.

In this paper, a combination of ion implantation technology and heat treatment is used to prepare a nonhomogeneous modified layer on the surface of 316 L stainless steel, which simultaneously meets the electrical conductivity and corrosion resistance requirements of stainless steel bipolar plates for commercialization. Because of the good electrical conductivity of Mo and C elements and the excellent corrosion resistance of molybdenum’s metal carbide, the strategy of heat treatment and Mo+C dual ion implantation was chosen. Mo+C ion implantation improves the elemental composition of the stainless steel surface and enhances the electrical conductivity of the stainless steel, and the subsequent heat treatment operation removes the damage caused by the radiation and alters the surface’s organizational structure [22,23], which results in the enhancement of the surface homogenization, and the corrosion resistance is improved. Therefore, this study aims to systematically investigate the impact of heat treatment on the electrical conductivity and corrosion resistance of Mo and C dual ion implanted 316 L stainless steel in a PEMFC environment using AFM, SEM-EDS, GD-OES, and XPS techniques.

## 2. Experimental Methods

### 2.1. Sample Preparation

The SS 316 L samples were small cylinders measuring 15 mm in diameter and 6 mm in thickness made of 316 L stainless steel. They underwent a gradual grinding and polishing process using SiC sandpaper as well as diamond abrasive paste to achieve a mirror finish. Subsequently, the SS 316 L samples were subjected to ultrasonic cleaning in acetone at room temperature for 20 min before being vacuum sealed for storage purposes. The SS 316 L samples were subjected to ionization using a Model 50 MEVVA (Beijing Normal University, Beijing, China) source metal vapor vacuum arc ion implanter, followed by ground and polishing. Mo^+^ and C^+^ ions were injected at accelerating voltages of 40 kV and 30 kV, with injection doses of 4.5 × 10^17^ ions/cm^2^ and 6 × 10^17^ ions/cm^2^, respectively. Subsequently, the ion implanted samples underwent heat treatment in an OTF-1200X-S miniature open tube furnace at temperatures of 600 °C, 800 °C, and 1000 °C for a holding time of 1 h each, which are denoted as (Mo+C)-600, (Mo+C)-800, and (Mo+C)-1000, respectively. The heat treatment process was conducted under Ar_2_ atmosphere and cooled within the furnace.

### 2.2. Surface Characterization

The surface morphology of stainless steel samples and modified samples was characterized using a dimension icon atomic force microscope (AFM) (Bruker Corporation, Billerica, MA, USA). Elemental distribution and content analysis of the samples on the surface were conducted using a scanning electron microscope-energy dispersive X-Ray spectrometer (SEM-EDS) (Carl Zeiss AG, Oberkochen, Germany). Depth profiling of elements in the modified layers was performed through analysis with a GDS850A glow discharge atomic emission spectrometer (GD-OES) (Laboratory Equipment Corporation, Joseph, MI, USA). Surface components of the samples were analyzed utilizing a Thermo Scientific K-Alpha X-ray photoelectron spectroscopy (XPS) (Thermo Fisher Scientific, Waltham, MA, USA) analyzer, with the binding energy spectrum fully corrected to the binding energy of the heterocyclic carbon peak at 284.8 eV.

### 2.3. ICR Measurements

**Figure 1:**
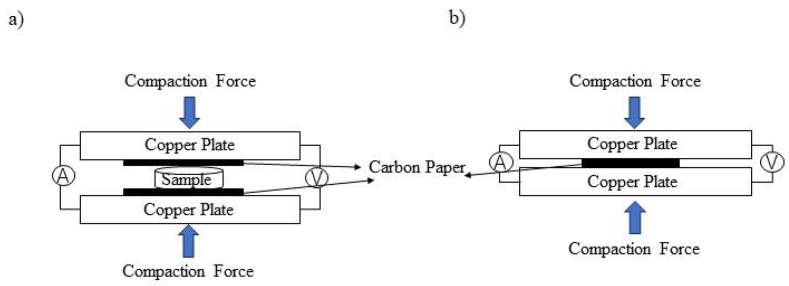
Schematic of the contact resistance measurement: (**a**) total resistance R_1_ (Cu|CP|sample|CP|Cu) and (**b**) R_2_ (Cu|CP|Cu).

The investigation of interfacial contact resistance (ICR) between the bipolar plate and carbon paper in PEMFC stacks is crucial. In this study, the sandwich method was employed to measure ICR before and after ion implantation, as well as for heat-treated samples [24,25,26]. The experimental setup is illustrated in Figure 1. The external part of the unit comprised two copper plates acting as clamping plates with soldered wires for current conduction. The sample was positioned between two sheets of Torry TGP-H-900 carbon paper, which were inserted between the parallel copper plates. By applying a constant current of 0.1 A across the parallel copper plates, a voltage was generated due to both material contact resistance and internal resistance within the materials themselves. A pressure ranging from 60 N to 300 N was applied at both ends of the device while recording voltage values at different pressures. The accuracy and reliability of the data were ensured by conducting three tests on each sample and calculating the average value.

### 2.4. Electrochemical Measurements

To expedite material screening and reduce experimental time, a more acidic corrosion solution, known as an accelerated corrosion solution, is typically employed for conducting corrosion resistance experiments on bipolar plates. Therefore, the simulated corrosion solution utilized in this study consisted of a 0.5 mol/L H_2_SO_4_ + 2 ppm F^−^ solution at a temperature of 80 °C. To simulate real working condition perturbations, an oil bath with stirring was used for heating purposes. The cathode was oxygenated at a rate of 20 mL/min. Electrochemical testing was conducted using a CHI 660E electrochemical workstation (Shanghai Chenhua Instrument Co., Shanghai, China) within a typical three electrode system. Prior to electrochemical testing, the samples underwent ultrasonic cleaning in acetone for 20 min followed by drying, and the cathodic potential was set at 0.6 V (SCE). The stability of samples in cathodic accelerated corrosion solutions was assessed using both potentiodynamic and potentiostatic polarization tests.

## 3. Results and Discussion

### 3.1. Characterization of the Modified Layer

**Figure 2:**
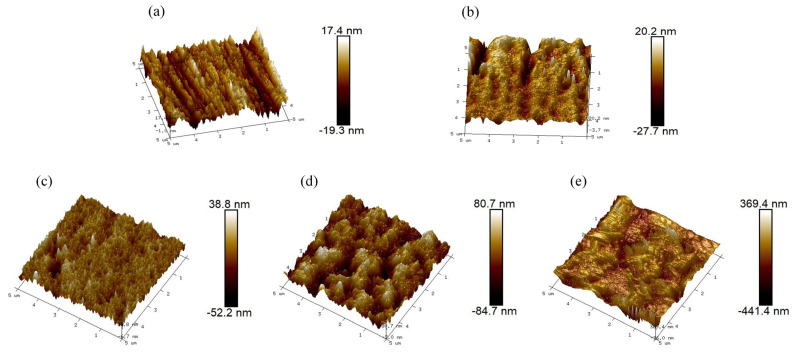
AFM diagram of SS 316 L under various modified conditions: (**a**) 316 L, (**b**) (Mo+C), (**c**) (Mo+C)-600, (**d**) (Mo+C)-800, (**e**) (Mo+C)-1000.

Figure 2 shows the surface morphology of SS 316 L bipolar plates modified by different methods. The pristine SS 316 L bipolar plate presents a smooth surface morphology with a surface roughness of 4.68 nm (Table 1). When the bipolar plate was implanted with Mo+C ions, the smooth surface was destroyed, and instead, uneven deep ravines were formed (Figure 2b). This discrepancy in surface morphology is related to the sputtering of the interaction between the ion beam and the elements of the substrate, which enlarged the original grooves and gaps of the SS 316 L substrate. After heat treatment at different temperatures, more ravines were distributed on the surface of the sample, and the roughness became larger still. However, the distribution of these ravines was more uniform than that of the ion-implanted sample, as shown in Figure 2c–e. In particular, the (Mo+C)-600 sample showed significant particle size refinement and homogenization of the surface material. The above situation occurred because high temperature heating will make the elements within the stainless steel combine with each other bringing precipitation to the surface, which both repairs the irradiation damage during ion injection and homogenizes the surface; the precipitated material is mainly metal carbides and oxides. Because of the injection of Mo and C double ions, the carbides formed by heat treatment will inhibit the precipitation of surface oxides to a certain extent, and the carbides precipitated from the surface are mainly Mo_2_C. Metal carbides have better stability, conductivity, and corrosion resistance compared to metal oxides.

**Figure 3:**
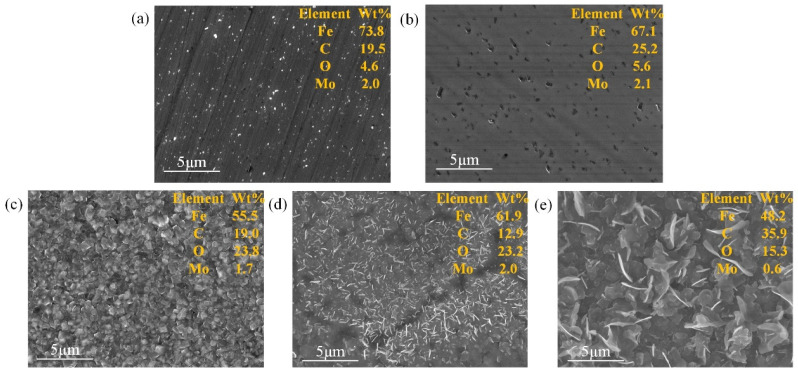
SEM of SS 316 L under different modification conditions: (**a**) 316 L, (**b**) (Mo+C), (**c**) (Mo+C)-600, (**d**) (Mo+C)-800, (**e**) (Mo+C)-1000.

Figure 3 shows the SEM images and the corresponding EDS results of the 316 L. With the bare 316 L (Figure 3a), the ion implanted sample (Figure 3b) increased the content of Mo and C elements on the surface, which is favorable for the improvement of surface conductivity. Moreover, due to the irradiation damage caused by the ion bombardment of the substrate surface during the ion implantation process, many holes were generated on the sample surface. The presence of holes, which are prone to localized corrosion under acidic conditions, can influence the corrosion resistance of the sample. From Figure 3c–e, it was found that as the heat treatment temperature increased, the grains of the material generated on the surface increased and thickened to repair the irradiation damage, which corresponded to the change in the surface roughness of the Figure 2 samples. With the heat treatment at 800 °C and 1000 °C, the scale-like metal oxides and hydroxides were generated so that the sample morphology had great variability; the higher the temperature the larger the grain. Too high a temperature is likely to lead to excessive growth of the grain, which has a certain effect on corrosion resistance. Refined and homogenized grains were obtained on the surface of (Mo+C)-600 samples, which facilitated the increase in corrosion resistance of the samples.

**Figure 4:**
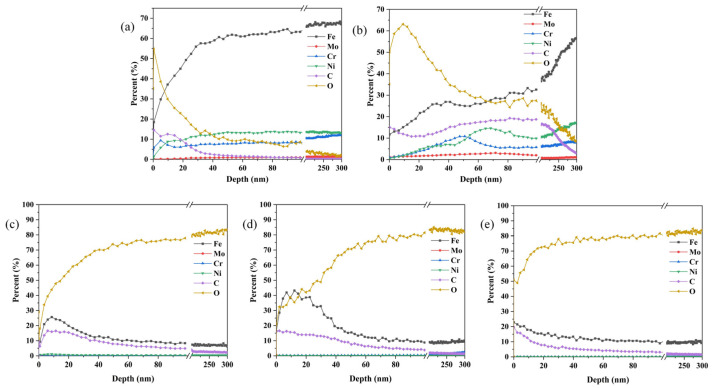
GD-OES of SS 316 L under different modification conditions: (**a**) 316 L, (**b**) (Mo+C), (**c**) (Mo+C)-600, (**d**) (Mo+C)-800, (**e**) (Mo+C)-1000.

Figure 4 shows the element distribution of different 316 L samples along the depth direction. An obvious variation of the elemental distribution occurred in different samples. For the pristine 316 L, the content of Fe, Cr, and Ni elements increased rapidly with depth and then remained almost unchanged, while the content of O and C elements decreased rapidly and then slowly. In the case of implanted sample, the ion implantation depth measured approximately 80 nm. The content of C element firstly decreased, then increased slowly, and then decreased rapidly after 200 nm; the content of Mo element firstly increased and then gradually decreased around 70 nm. The Fe content on the surface of the substrate decreased by 30%, the Cr and Ni content slightly decreased, and the O content increased by 20–30%. The elevated O element content indicates that the surface was partially oxidized during dual ion implantation when the ions were sputtered for a long time and the substrates collided with each other to produce a high temperature of 100–200 °C. After heat treatment, the elements near the surface of the samples were mainly Fe, C, and O. The content of O increased rapidly with depth, and remained basically unchanged after 40 nm, with an overall decreasing trend in the content of Fe and C elements. With the increase of heat treatment temperature, C and O elements came to the interface of the aggregation precipitation, and other metals combined with the generation of metal carbides and metal oxides. The Mo content decreased dramatically at a heat treatment temperature of 1000 °C, insulated for 1 h, when the surface Mo content basically fell to 0, mainly because of a large number of iron oxides and a small amount of cementite on the surface and covering the molybdenum element.

**Figure 5:**
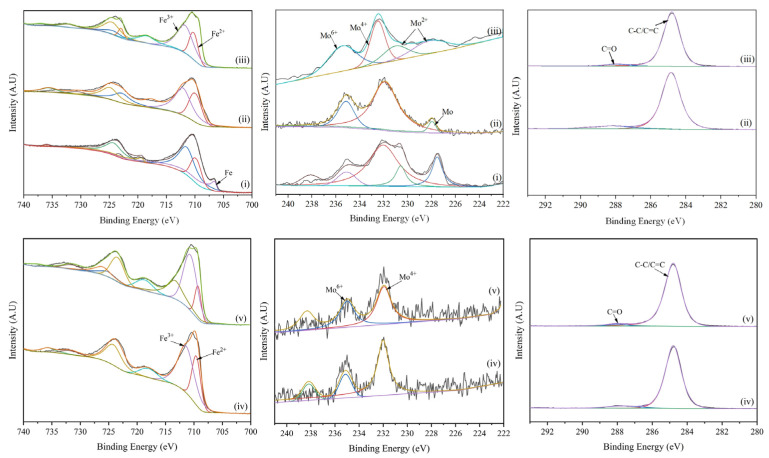
XPS spectra of Fe, Mo and C elements on the surface of different modified samples (i) 316 L, (ii) (Mo+C), (iii) (Mo+C)-600, (iv) (Mo+C)-800 and (v) (Mo+C)-1000.

XPS analysis was performed to gain an insight into the chemical state of elements on the sample surface. Figure 5 shows the Fe 2p, Mo 3d, and C 1s XPS spectra of 316 L stainless steel under different modification conditions. Each Fe spectrum contains two main peaks (~710 eV for Fe 2p_3/2_ and ~725 eV for Fe 2p_1/2_). For the pristine 316 L stainless steel, three components were observed in the deconvoluted Fe 2p spectra. The Fe 2p_3/2_ peak at 706.6 eV could be assigned to Fe in the 316 L stainless steel phase. In the case of the ion implanted sample, the surface oxygen content is increased, so Fe monomers were oxidized to Fe^2+^ (Fe 2p_3/2_, 710 eV) and Fe^3+^ (Fe 2p_3/2_, 712 eV). In the Mo 3d spectra of the ion implanted samples, the Mo 3d_5/2_ peak at 227.9 eV is monomeric Mo, and the binding energy of the Mo monomers became higher after ion implantation. Mo can improve surface conductivity.

As shown in Figure 5(iii), the main peaks of the (Mo+C)-600 samples Fe 2p_3/2_ and Fe 2p_1/2_ are shifted toward higher binding energies. The Mo 3d peak fit shows that Mo existed in three states (2+, 4+ and 6+) in the sample surface. The peaks observed at binding energies of 232.49 eV (Mo 3d_5/2_) and 235.50 eV (Mo 3d_5/2_) can be attributed to Mo^4+^ and Mo^6+^, respectively, which may arise from either oxygen migration within the sample during high temperature treatment at 600 °C or surface oxidation upon exposure to air [27,28]. Additionally, characteristic double peaks located at binding energies of 228.2 eV (Mo 3d_3/2_) and 231.1 eV (Mo 3d_5/2_) correspond well with those reported in the literature for Mo_2_C’s Mo^2+^ state [29,30]. These findings demonstrate that heating at a temperature of 600 °C followed by a holding time of one hour leads to precipitation of Mo carbides on stainless steel substrate surfaces, thereby enhancing their conductivity and corrosion resistance [31].

### 3.2. Interfacial Contact Resistance

**Figure 6:**
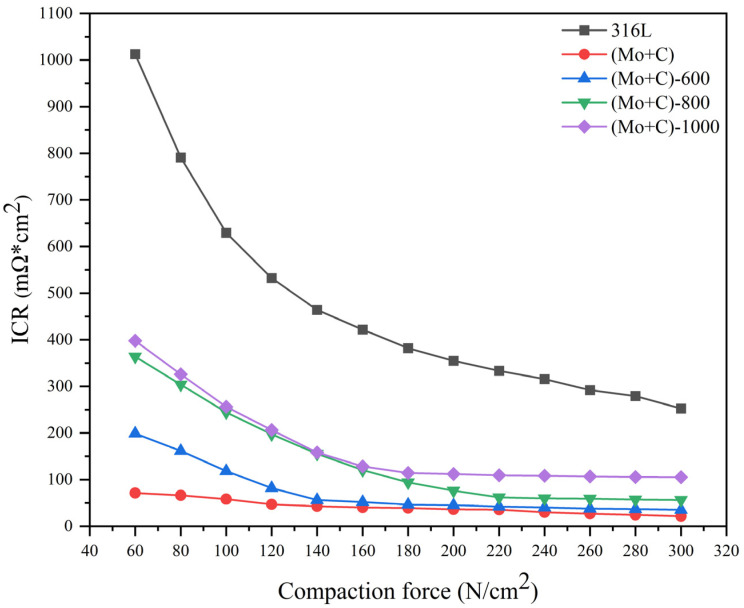
Interfacial contact resistance between carbon paper and under different modification conditions SS 316 L BPs as a function of compaction force.

The interfacial contact resistance (ICR) serves as a crucial performance indicator for fuel cells, and the results are presented in Figure 6. Both bare 316 L and samples subjected to various modification conditions exhibited similar trends with increasing compressive force. Initially, the ICR decreased rapidly within the low pressure range of 60–140 N/cm^2^, followed by a slower decrease until reaching stabilization when the compressive force exceeded 200 N/cm^2^. This behavior can be attributed to the limited actual contact area between the sample and carbon paper under low pressure conditions, resulting in poor conductivity and higher ICR values. As pressure continued to increase, deformation of the carbon paper enhanced contact points with the sample, thereby providing more conduction paths and reducing the ICR value. At an assembly force of 140 N/cm^2^, bare 316 L displayed an ICR value of 464 mΩ × cm^2^ compared to only 42 mΩ × cm^2^ for Mo and C dual ion implanted samples—a remarkable reduction in ICR of approximately 91%. The results demonstrate that the dual injection of Mo and C ions positively contributes to enhancing the electrical conductivity of bipolar plates. The ICR values of the samples increased with increasing heat treatment temperature but remained significantly lower than those of bare SS 316 L, showing a decrease by 64–86%. The higher ICR values in heat-treated samples compared to ion implanted samples can be attributed primarily to an increase in surface oxygen content and metal oxides resulting from heat treatment. However, the thickening of the surface passivation layer adversely affected the electrical conductivity of bipolar plates, leading to elevated ICR. As shown in Figure 4, although there was an increase in surface oxygen content with rising heat treatment temperature, it consistently remained lower than that observed on bare 316 L surfaces. Moreover, as temperature increased, carbon accumulated at the surface causing much lower ICR values for heat treated samples compared to bare 316 L. Additionally, as the temperature rose further, there was a more rapid increase in surface oxygen content; hence higher temperatures resulted in higher ICR values on sample surfaces. The (Mo+C)-600 samples showed a large difference in ICR values in the range of 60–120 N/cm^2^ compared to the ion implanted samples, but the ICR values were essentially the same at the actual assembly force of 140 N/cm^2^.

### 3.3. Potentiodynamic Polarization Test

**Figure 7:**
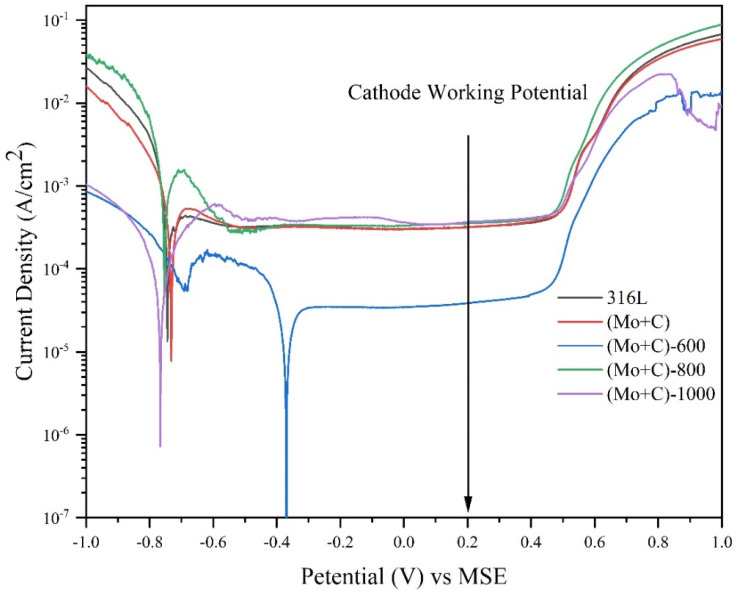
Tafel curves obtained from the polarization test carried out in simulated operating environments of cathode for different samples.

The potentiodynamic polarization curves of each sample in a cathodic environment are depicted in Figure 7. The corrosion data (E_corr_(V) is the corrosion potential; I_corr_(A/cm^2^) is the corrosion current density; E_tran_(V) is the transpassivation potential) obtained from the fitting of potentiodynamic polarization curves are shown in Table 2. Each SS 316 L sample exhibited a typical corrosion profile for stainless steel with a self-passivation behavior in accelerated corrosion solutions, with all samples showing wide passivation intervals from the self-corrosion potential onwards. The corrosion potentials of bare 316 L and each modified sample were in the range of −0.8 V~−0.3 V (MSE) under cathodic conditions, shown in Figure 7. The bare 316 L had a corrosion potential of around −0.743 V (MSE). Both (Mo+C) and (Mo+C)-600 samples exhibited elevated corrosion potentials compared with 316 L samples; particularly noteworthy was that the (Mo+C)-600 sample demonstrated a self-corrosion potential of around −0.369 V (MSE) which surpasses 316 L samples significantly. The branching peaks of (Mo+C)-600 samples may be related to the reduction of passivation films (MoO_2_, MoO_3_). This reduction resulted in a change in the state of the electrode surface, which led to a change in the hydrogen evolution reaction process. This increase in corrosion potential signified enhanced chemical inertness of the sample surface and indicated improved stability within corrosive environments.

The corrosion current densities of several samples were calculated by fitting, and the (Mo+C)-600 sample had the smallest corrosion current density (5.09 × 10^−5^ A/cm^2^) among all the samples. The corrosion current density was significantly smaller than the 316 L sample, and the corrosion current density was smaller than the 316 L sample by two orders of magnitude from the results in Table 2. This is mainly due to the generation of metal carbides on the surface of the (Mo+C)-600 samples which improves the corrosion resistance of the surface. The corrosion current density of the ion implanted sample was comparable to the 316 L sample. This could be attributed to the creation of surface defects and irradiation damage caused by ion implantation, and this also corresponded to the AFM and SEM test results. The stability of the passivation film formed on the surface was compromised, leading to a deterioration in corrosion.

The corrosion current density and corrosion potential of the (Mo+C)-600 sample were much smaller than the other modified samples. These findings highlighted that heat treatment at 600 °C effectively mitigated corrosive attack on bipolar plates made from 316 L stainless steel.

### 3.4. Potentiostatic Curves of SS 316 L Substrate and Modified Samples

**Figure 8:**
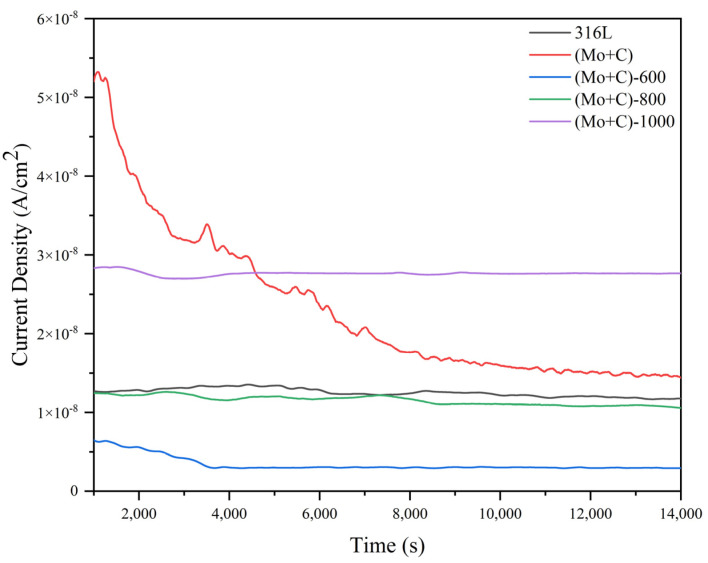
Potentiostatic curves of SS 316 L substrate and modified samples in simulated operating environments of cathode.

The constant potential current-time curve (I-t curve) was employed to assess the reliability and stability of bare 316 L and each modified sample under accelerated corrosion conditions for extended operation periods. Figure 8 illustrates the electrostatic potential current-time curves for each sample in the simulated cathodic accelerated corrosion environment. After 4 h of electrostatic potential polarization, all samples except (Mo+C) exhibited gradually stabilizing corrosion current densities, indicating that dissolution and passivation film generation reached dynamic equilibrium with minimal required corrosion current density to maintain relative passivation film stability.

At the same time, the (Mo+C)-600 demonstrated significantly lower corrosion current density levels compared to unmodified 316 L samples, as shown in Figure 8. The corrosion current density was reduced from 1.21 × 10^−8^ A/cm^2^ to 2.95 × 10^−9^ A/cm^2^, and the corrosion resistance of the (Mo+C)-600 sample was improved by 75.62%. The conclusion could be drawn that carbides and oxides generated through surface precipitation could effectively inhibit sample surface corrosion and enhance overall resistance against corrosive environments when subjected to heat treatment conditions at high temperatures of 600 °C.

## 4. Conclusions

The nonhomogeneous modified layers were prepared on SS 316 L surfaces using ion implantation and heat treatment methods. These samples were successfully utilized in PEMFC bipolar plates for testing electrical conductivity and corrosion resistance. In comparison to bare 316 L, the injection of Mo and C double ions induces surface irradiation damage, resulting in significantly improved electrical conductivity but not a substantial enhancement in corrosion resistance. However, this ion induced irradiation damage can be repaired through heat treatment by precipitating carbides on the stainless steel surface, particularly when heated at 600 °C for 1 h. Metal carbide exhibits high electrical conductivity and corrosion resistance properties. (Mo+C)-600 bonds well to the substrate without any surface or interfacial defects such as microcracks. Moreover, considering its excellent surface conductivity and corrosion resistance characteristics, the (Mo+C)-600 sample is considered a promising candidate material for bipolar plates. Under a compressive force of 140 N/cm^2^, the interfacial contact resistance value of (Mo+C)-600 is basically similar to that of the ion implanted sample and much lower than that of untreated 316 L (464 mΩ × cm^2^). Electrochemical experiments demonstrate that under simulated PEMFC cathodic ambient conditions, (Mo+C)-600 samples exhibit superior passivation behavior and considerably higher corrosion resistance compared to untreated 316 L. Therefore, it can be concluded that (Mo+C)-600 holds great potential as a prospective bipolar plate material for PEMFC.

## Figures and Tables

**Table 1:** Surface roughness of 316 L stainless steel samples under different modification conditions.

Sample Code	Surface Roughness (Image Ra, nm)	Surface Roughness (Image Rq, nm)
316 L	4.68	5.87
(Mo+C)	5.38	7.70
(Mo+C)-600	6.87	8.96
(Mo+C)-800	23.20	28.80
(Mo+C)-1000	100.00	127.00

**Table 2:** The results of polarization test for different samples in cathodic simulation conditions.

Samples	E_corr_ (V)	I_corr_ (A/cm^2^)	E_tran_ (V)
316 L	−0.743	7.01 × 10^−3^	0.484
(Mo+C)	−0.731	5.33 × 10^−3^	0.494
(Mo+C)-600	−0.369	5.09 × 10^−5^	0.462
(Mo+C)-800	−0.747	13.84 × 10^−3^	0.477
(Mo+C)-1000	−0.766	4.08 × 10^−4^	0.497

## Data Availability

Data are contained within the article.
